# Different treatment strategies for acromioclavicular dislocation injuries: a nationwide survey on open/minimally invasive and arthroscopic concepts

**DOI:** 10.1186/s40001-019-0376-7

**Published:** 2019-03-23

**Authors:** F. Allemann, S. Halvachizadeh, M. Waldburger, F. Schaefer, C. Pothmann, H. C. Pape, T. Rauer

**Affiliations:** 1 Department of Trauma, University Hospital Zurich, Raemistrasse 100, 8091 Zurich, Switzerland; 2 Medical School, University of Zurich, Zurich, Switzerland

**Keywords:** Acute acromioclavicular separation, Arthroscopically assisted acromioclavicular joint stabilization, Hook plate stabilization, Rockwood typ III lesion

## Abstract

**Background:**

Injuries to the acromioclavicular (AC) joint are one of the most common among sporting injuries of the upper extremity. Several studies investigated different treatment options comparing surgical and non-surgical treatment, and type of operative interventions. This study aims to evaluate treatment decisions regarding injuries of the AC joint and to compare different treatment strategies depending on the specific training of the treating physician.

**Methods:**

We performed a nationwide survey by contacting different experienced physicians and sending them questionnaires. The questionnaire included 37 questions that assessed preferred treatment strategies in AC joint injuries. We addressed different surgical and nonsurgical options as well as level of experience and factors that might influence the decision on treatment strategy. The physicians were stratified according to their training into general surgeons (group trauma associated) and orthopedic surgeons (orthopedic associated). The AC joint lesions were categorized according to the widely used Rockwood classification.

**Results:**

This study analyses 96 questionnaires. We included 46 (47.9%) colleagues in group trauma and 50 (52.1%) in group orthopedics. Most of the colleagues (98.9%) prefer non-operative treatment of type I and type II AC lesions. Similarly, 96.8% agree on surgical treatment of types IV, V, and VI lesions. The treatment of type III lesions is performed in 41.6% of cases non-operatively and in 58.4% of cases surgically. Trauma-associated colleagues are 3.4 times more likely to treat AC lesions with a hook plate compared to orthopedic-associated colleagues (*p* = 0.05). In decreasing order, the most commonly used non-surgical technique is sling immobilization (63.7%), and the most commonly performed surgical treatment is the hook plate (41.1%) in treating type III injuries.

**Conclusion:**

This study shows a distinct difference in treatment of AC joint injuries depending on the training of the physician. Further, the need for high-quality studies arises to define the optimal treatment of type III lesions.

## Background

Acromioclavicular (AC) dislocations are frequent injuries and are often sports related. Among American football players, of which 50% describe shoulder injuries, the most common injuries are AC separations [1]. Depending on the number of injured ligaments, AC dislocations are classified based on severity. AC dislocations are classified by Tossy and Allman [2, 3] into three types: in type I lesions, the AC ligaments are sprained; in type II lesions, those ligaments are ruptured; and in type III lesions, the distal clavicle is horizontally and vertically unstable. Rockwood [4] modified this classification by adding three more types: a type IV lesion, which is defined as a rupture of the AC and the coracoclavicular (CC) ligaments as well as the deltopectoral fascia; a type V lesion with significant displacement of the AC joint, i.e., with an 100–300% increased CC distance; and an exceedingly rare type VI lesion, in which the distal clavicle is inferiorly displaced into a subacromial or subcoracoid position behind the biceps tendon. The ISAKOS (International Society of Arthroscopy, Knee Surgery & Orthopedic Sports Medicine) recommended a further refinement by subclassifying the Tossy/Rockwood type III lesion into a stable type IIIA and an unstable type IIIB lesion [5]. Patients with a type IIIB lesion show significant weakness of the rotator cuff and pain during clinical examination. Furthermore, these patients will have a limited abduction and flexion of the shoulder joint [6].

Conservative treatment is the commonly recommended treatment option for types I and II lesions. With respect to Rockwood types IV–VI lesions, operative treatment is clearly recommended.

In contrast, non-operative treatment of type III lesions has not shown satisfactory results and such injuries are, therefore, often surgically treated. Different fixation options have been described, including tension band wiring [7].Most of these surgical treatment options have, however, been abandoned due to increased complication rates as well as suboptimal results. However, open reduction and internal fixation (ORIF) with Balser hook plating showed good results in type III injuries [8], but hardware removal is often necessary due to discomfort. Further, several studies reported high postoperative recurrence rates with loss of anatomical reduction over time [9–11]. Additionally, some studies [6] could not prove any significant differences in pain or rate of posttraumatic, regardless of the treatment option. The functional outcome in both conservative and surgical treatment options is also the subject of current research. Therefore, treatment of type III lesions remains controversial in the literature [5, 12].

Newer, less invasive techniques with promising initial results have been developed. For example, MINAR (minimally invasive AC joint reconstruction) is a commonly used, minimally invasive open treatment option in which the coracoid process is exposed by a minimal incision. A hole is drilled through the coracoid process with the help of a specific aiming device. The suture cerclage is connected to two buttons. One of the buttons is then pushed through the coracoid process. The button is flipped and the suture is thereby fixed to the coracoid process. The other anchor is pulled through a hole in the clavicle and the cerclage is secured with a surgical knot after reduction of the AC joint. Further, promising arthroscopic button techniques have also been developed.

We are convinced that the varying treatment options for type III lesions should be further discussed and we, therefore, developed a nationwide Swiss survey. The primary objective of this study is to evaluate currently preferred treatment options and the reasoning behind them in Rockwood type III lesions. Secondarily, we evaluated the different surgical treatment concepts currently utilized in type III lesions.

## Materials and methods

A nationwide survey in Switzerland was conducted using an online questionnaire. The complete survey consisted of 37 questions assessing diagnosis and treatment of acromioclavicular injuries. The questionnaire consisted of 5 blocks. The first block assessed the hospital level, surgical specialization and surgical experience. The second block obtained information regarding diagnostics. The third and fourth blocks each included 7 questions on the treatment of type I/II and types IV/V and VI lesions. Finally, the fifth block consisted of 9 questions assessing treatment considerations of type III lesions.

With the goal of obtaining about 100 completed questionnaires, nearly 1000 online surveys were sent either to trauma surgeons or orthopedists.

### Statistics

Statistical analysis was performed using R (R Core Team (2018). R: A language and environment for statistical computing. R Foundation for Statistical Computing, Vienna, Austria. URL: https://www.R-project.org/). The data were tested for normality using the Shapiro Test. ANOVA was used to compare groups on continuously scaled variables with a normal distribution. Kruskal–Wallis test was used for non-normally distributed continuous variables with a skewed distribution. The Pearson *χ*^2^-test was applied to compare groups on categorical variables. Graphs were plotted using GraphPad Prism version 8.00 for Windows, GraphPad Software, La Jolla California USA, http://www.graphpad.com. Significance level was set at a *p* value ≤ 0.05 (two-sided).

## Results

A total of 138 questionnaires were received during a 2-month period in 2018. Of the 138 completed questionnaires, 96 (69.6%) were complete and 42 (30.4%) were incomplete. The evaluation included only the completed questionnaires.

Of the physicians completing the survey, 50 surgeons were Foederatio Medicorum Helveticorum (FMH) board-certified general surgeons with or without sub-specialization in trauma surgery (FMH trauma surgeon group, *n* = 50). The remaining surveys were completed by FMH or Swiss-equivalent board-certified orthopedic surgeons (orthopedic surgeon group, *n* = 46).

### Treatment preferences

Preferred treatment for Rockwood type I/II lesions was non-operative in 98.9% of cases. Only one participant (1%) in the trauma surgeon group preferred surgical treatment. In 96.8% of cases, types IV–VI lesions are treated operatively, while 3.1% of surveyed surgeons prefer conservative treatment.

In type III lesions, non-operative care is performed by 41.6% of surgeons while 58.3% of surgeons operate. However, subjectively, only 41.7% of surveyed surgeons preferred operative treatment (*n* = 40), while 31.2% preferred non-operative treatment (*n* = 30), and 26 surgeons (27.1%) expressed no personal preference between operative and non-operative treatment of Rockwood type III lesions.

**Fig. 1:**
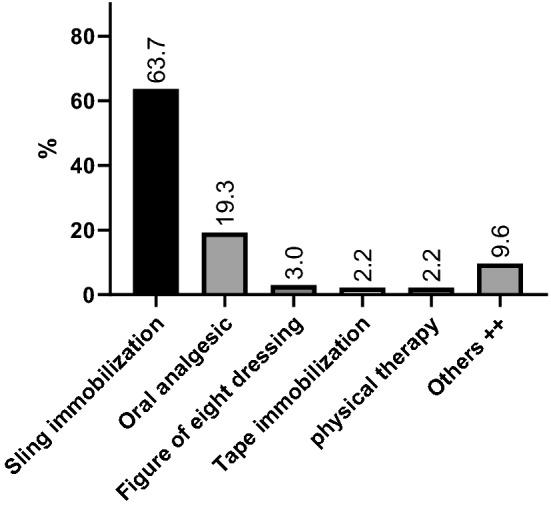
The most preferred treatment of types I, II and III is sling mobilization, followed by no immobilization combined with oral analgesic treatment. ^++^Patient did not want treatment or received other bandages

Preferred conservative treatment for type III lesions consisted of sling immobilization in 70%, oral analgesic medication in 10%, figure of eight bandage in 5%, tape immobilization in 2.5%, and physical therapy in 2.5% of surveyed surgeons, while 10% preferred other immobilization techniques or no specific treatment (*p* ≤ 0.001, *n* = 40). Preferred non-operative treatments are summarized in Table 1/Fig. 1.

**Table 1:** Summary of preferred non-operative treatment of AC joint injuries (types I, II and III lesions)

	Types I/II	Type III	OR	95% CI	*p*
	*n* = 95, %	*n* = 40, %			
Non-operative treatment					
Sling immobilization	61.1	70	0.7	0.3–1.5	ns
Tape immobilization	2.1	2.5	0.8	0.07–9.5	ns
Oral analgesic	23.2	10	2.7	0.8–8.5	ns
Physical therapy	2.1	2.5	0.8	0.07–9.5	ns
Figure of eight dressing	2.1	5	0.4	0.05–3.0	ns
Others	9.5	10	0.9	0.3–3.2	ns

The most preferred treatment is sling mobilization, followed by no immobilization combined with oral analgesic treatment. No significant differences between the groups types I/II and type III were found

**Fig. 2:**
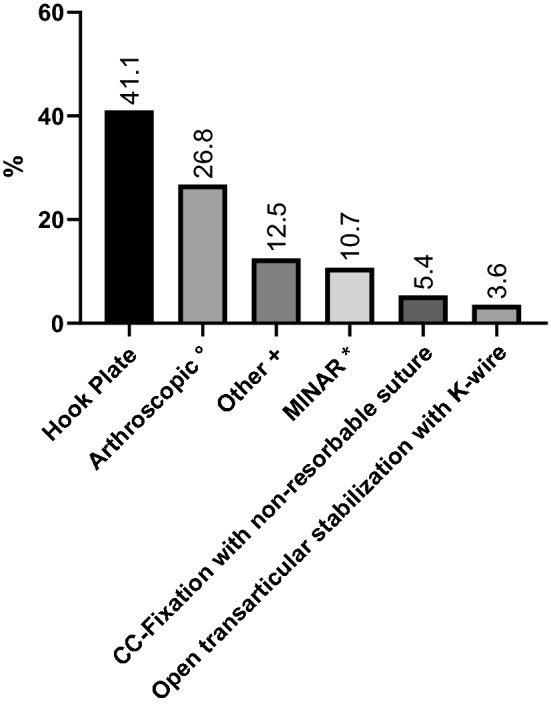
Preferred operative treatment of type III lesions. °Dog Bone Button Technology (Arthrex Inc., Naples Florida USA). *MINAR (minimally invasive AC joint reconstruction): The coracoid process is exposed by a 3-cm-long skin incision. A hole is drilled through the coracoid process with the help of a specific aiming device. The suture cerclage is connected to two buttons. One of the buttons is then pushed through the coracoid process. The button is flipped and the suture is thereby fixed to the coracoid process. The other anchor is pulled through a hole in the clavicle and the cerclage is secured with a surgical knot after reduction of the AC joint. ^+^Others including Hook plate combined with CC stabilization/Mitek anchor/extended capsule fixation; BIPOD technique (arthroscopic repair CC and AC ligaments to achieve bidirectional stability), and other combinations according to the individual in-hospital protocol

Preferred surgical treatment of type III lesions is summarized in Fig. 2. Hook plating was the most common procedure with 41.1% of surgeons preferring this modality. An arthroscopic technique surgery utilizing Dog Bone Button Technology (Arthrex Inc., Naples Florida USA) was also common, being utilized by 26.8% of surveyed surgeons; while the minimally invasive MINAR technique was preferred by approximately 10.7% of surgeons. Approximately, one quarter of surveyed surgeons used other techniques including Mitek anchor fixation of the coracoclavicular ligaments with or without additional hook plating. About 3% of surgeons still prefer tension banding of the AC joint.

Further, the survey assessed which external factors helped surgeons to indicate surgery in type III lesions. A majority of surgeons stated that manual overhead labor (*n* = 53, 55%) or overhead sports activities (*n* = 60, 63%) helped to steer surgeons towards operative treatment modalities. For 48 (49.5%) of surgeons, younger patients between 20 and 40 years of age were a further important external factor supporting operative reconstruction. Most surgeons (*n* = 88, 93%) stated that patients aged 41–65 years had no impact on justifying operative care (*p* ≤ 0.001). Additionally, 97% (*n* = 92) of surveyed surgeons stated that surgery in patients aged greater than 65 years would not have a positive impact on outcome (*p* ≤ 0.001). Twenty-two (22.7%) surgeons felt that the assessed external factors do not play a role in the indication for surgical treatment (Table 2).

**Table 2:** Categories that were described as having an impact on the decision to operate

Category	%^a^	Pearson *χ*^2^
Manual laborer overhead	27.5	ns
Over-the-head sports activities	31.1	0.01
20–40 years	24.9	ns
41–65 years	3.6	< 0.0001
> 65 years	1.6	< 0.0001
No factors	11.4	< 0.0001

Significant differences were found in age and in overhead sports activities

^a^The percentages are based on the possibility of multiple answers to a total of 193 statements

A significantly higher rate of participants favors the operative treatment of types IV–VI lesions (*n* = 93, 96.9%) over non-operative treatment (*n* = 3, 3.1%, *p* value ≤ 0.001).

**Fig. 3:**
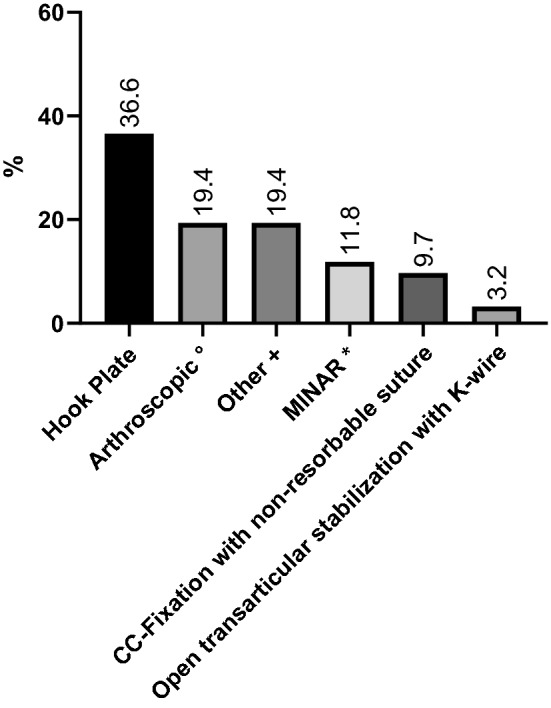
Preferred operative treatment options of types IV, V and VI lesions. °Dog Bone Button Technology (Arthrex Inc., Naples Florida USA). *MINAR (minimally invasive AC joint reconstruction): The coracoid process is exposed by a 3-cm-long skin incision. A hole is drilled through the coracoid process with the help of a specific aiming device. The suture cerclage is connected to two buttons. One of the buttons is then pushed through the coracoid process. The button is flipped and the suture is thereby fixed to the coracoid process. The other anchor is pulled through a hole in the clavicle and the cerclage is secured with a surgical knot after reduction of the AC joint. ^+^Others including Hook plate combined with CC stabilization/Mitek anchor/extended capsule fixation; BIPOD technique (arthroscopic repair CC and AC ligaments to achieve bidirectional stability), and other combinations according to the individual in-hospital protocol

Figure 3 displays the most favored operative techniques of treating types IV–VI lesions.

Finally, the preferred surgical techniques between the trauma surgeon and orthopedic surgeon groups were compared. Trauma-associated colleagues were significantly more likely to treat AC injuries of type III lesions with a hook plate compared to the orthopedic-associated colleagues (OR 3.4, 95% CI 1.1–11.3, *p* = 0.05). Additionally, several trends that did not show statistical significance were seen with more orthopedic specialists preferring arthroscopic techniques (38.1 vs. 20.0%) and MINAR more commonly utilized by trauma surgeons (14.3 vs. 4.8%) (Table 3).

**Table 3:** Overview about operative options in more trauma-associated and orthopedic-associated surgeons

	Trauma-associated colleagues *n* = 35, %	Orthopedic-associated colleagues *n* = 21, %	OR	95% CI	*p*
Arthroscopic technique^a^	20	38.1	0.4	0.1–1.4	ns
CC fixation with non-resorbable suture	5.7	4.8	1.2	0.1–14.2	ns
Hook plate	51.4	23.8	3.4	1.1–11.3	0.05
MINAR^b^	14.3	4.8	3.3	0.4–30.7	ns
Open transarticular stabilization with K-wire	2.9	4.8	0.6	0.03–9.9	ns
Others^c^	5.7	23.8	0.2	0.03–1.1	ns

^a^Dog Bone Button Technology (Arthrex Inc., Naples Florida USA)

^b^MINAR (minimally invasive AC joint reconstruction): The coracoid process is exposed by a 3-cm-long skin incision. A hole is drilled through the coracoid process with the help of a specific aiming device. The suture cerclage is connected to two buttons. One of the buttons is then pushed through the coracoid process. The button is flipped and the suture is thereby fixed to the coracoid process. The other anchor is pulled through a hole in the clavicle and the cerclage is secured with a surgical knot after reduction of the AC joint

^c^Hook plate combined with CC stabilization with Mitek anchor and an extended capsule fixation

## Discussion

AC separation is a common injury of the shoulder. The literature clearly shows that low-grade Rockwood types I–II lesions are best managed conservatively. The data in this survey correlate well with 99% of surgeons choosing conservative treatment [13]. For high-grade AC injuries, operative treatment is clearly supported in the literature. This nationwide survey again correlates with over 96% of surgeons preferring surgical care. Consistent with Tauber et al. [14], this approach is the preferred treatment option of trauma and orthopedic surgeons.

In contrast, treatment recommendations for type III lesions are not conclusive. Both conservative and surgical treatment strategies have been advocated [6]. Further, when choosing surgical care, no surgical gold standard exists. This national survey showed that 58% of surgeons would treat type III lesions surgically. Open, minimally invasive and also arthroscopic techniques have both advantages and disadvantages. In Europe, ORIF using a hook plate, AC reconstruction using MINAR technology as well as arthroscopic interventions have prevailed.

In our study, hook plate osteosynthesis and arthroscopic procedures were most commonly used with 41.1% and 26.8% of surgeons using these techniques, respectively. The current literature shows no significant clinical differences in outcome, but a tendency toward better results and higher patient acceptance is seen with arthroscopic procedures [15–17]. Stein et al. have also recently shown improved Taft and Constant scores 2 years postoperatively in patients with high-grade AC separations treated either arthroscopically with a CC-stabilizing double button suture or hook-plating. Further, CC stabilization showed decreased rates of persistent horizontal instability vs. patients treated with a hook plate [18]. Arirachakaran et al. showed that hook plate fixation have lower functional shoulder scores and higher postoperative pain when compared with a loop suspensory fixation [19]. However, hook plates may be combined with ligament reconstruction. For example, Yin et al. concluded that hook plating combined with a double-tunnel CC reconstruction showed superior results to hook plating alone [20]. Although hook plate osteosynthesis is a simple procedure, a second procedure for hardware removal is required. This treatment option is, therefore, quite expensive and arthroscopic procedures are significantly less expensive.

In addition, hook plating has been associated with an increased risk of recurrence with some studies showing a recurrence rate of 2.9% after removal of the plate [15]. In contrast, the MADOK AC reconstruction technique has shown recurrence rates as low as 0% [21]. This procedure uses an allograft sling with reinforcing internal sutures passed around the coracoid and passed through the clavicle for an anatomical CC reconstruction. In addition, the superior AC ligaments are reconstructed with a docking mechanism, with allograft reconstruction of the native AC ligaments.

The MINAR technique is used by 6.2% of surgeons surveyed and CC cerclage with non-resorbable materials are utilized by 3.1% of surgeons. Patient satisfaction and postoperative pain levels are lower with suture rather than metal implants. Darabos et al. [22], for example, showed greater satisfaction and lower discomfort with AC suture reconstruction than with Bosworth screw osteosynthesis. While Bosworth screws are no longer used in isolation, they are still sometimes combined with other treatment modalities. Tiefenboeck et al. [23] showed that Bosworth screw fixation with additional K-wire stabilization offers good-to-excellent functional outcomes and was well tolerated.

Open transarticular stabilization of the AC joint with and without a cerclage was reported as a preferred treatment by 2.1% of surgeons. While Murphy et al. [24] showed good postoperative stability and range of motion, tension banding and other k-wire transfixation have fallen out of favor due to high complication rates, including K-wire migration [25, 26]. Further, this treatment modality has probably also fallen out of favor due to the availability of better implants and development of newer techniques.

This survey also assessed external factors that support a surgical indication for type III lesions. The most common factors were manual overhead labor or overhead sports activities. In this survey, the majority of surgeons preferred operative treatment of type III lesions. Bajnar et al. also highlighted this tendency [27]. Recent literature also shows that patients treated with double suture button reconstruction were much more likely to return to previous sport activity levels compared to patients treated with hook plating [28].

Half of the surgeons surveyed in this study stated that patient age between 20 and 40 years was a decisive indication for surgery in type III lesions as younger patients are more physically demanding. Patient over 40 years old were not considered as an important indicator for surgery in this survey. With increasing age, both the surgical risk and the comorbidities increase, which probably play an important role in choosing conservative treatment. Presumably, a more defensive attitude towards surgical intervention in older patients may also be a factor.

Due to the complex ligamentous anatomy of the AC joint, a gold standard surgical technique is still elusive. Due to the large number of surgical techniques and extensive research in the field, we assume that no ideal treatment modality exists as no study has been able to show one modality with obviously superior outcomes. The variety of surgical techniques used to treat AC separation in this survey is most likely a result of these factors, but individual training and personal preferences may also play a role. For example, trauma and orthopedic surgeons used different surgical treatment modalities in this study. Half of the trauma surgeons preferred stabilization with hook plate osteosynthesis, while 38% of orthopedic surgeons preferred arthroscopic modalities. This study clearly shows that Swiss surgeons utilize many treatment options for AC joint separation. As the range of modern techniques including arthroscopic or minimally invasive surgical techniques becomes wider, the outcomes of specific procedures need to be further evaluated to finally find an optimal implant and surgical technique to further benefit patients and improve long-term outcomes.
